# Book Review: Principles and Practice of Burn Care

**DOI:** 10.1055/s-0045-1802645

**Published:** 2025-02-10

**Authors:** Dinesh Kadam

**Affiliations:** 1 Department of Plastic and Reconstructive Surgery, A. J. Institute of Medical Sciences and Research Centre, Mangalore, Karnataka, India

## Title: Principles and Practice of Burn Care

**Chief Editor: Sujata Sarabahi**

**Co-editors:** Dr V K Tiwari, Dr Arun Goel and Dr Suvashish Dash

**2 ^nd^ Edition, 2025**

**Publisher: Jaypee Brothers Medical Publishers (P) Ltd., New Delhi**

**Number of pages: 720**

I am delighted to review the second edition of “ *Principles and Practice of Burn Care* ” edited by Dr. Sujata Sarabahi along with Dr. V. K. Tiwari, Dr. Arun Goel, and Dr. Suvashish Dash. 1 First published in 2010, this comprehensive volume was the country's first comprehensive textbook on burns. It remains the foremost reference on burn management in India and remains an essential resource globally.

**Fig. 1:**
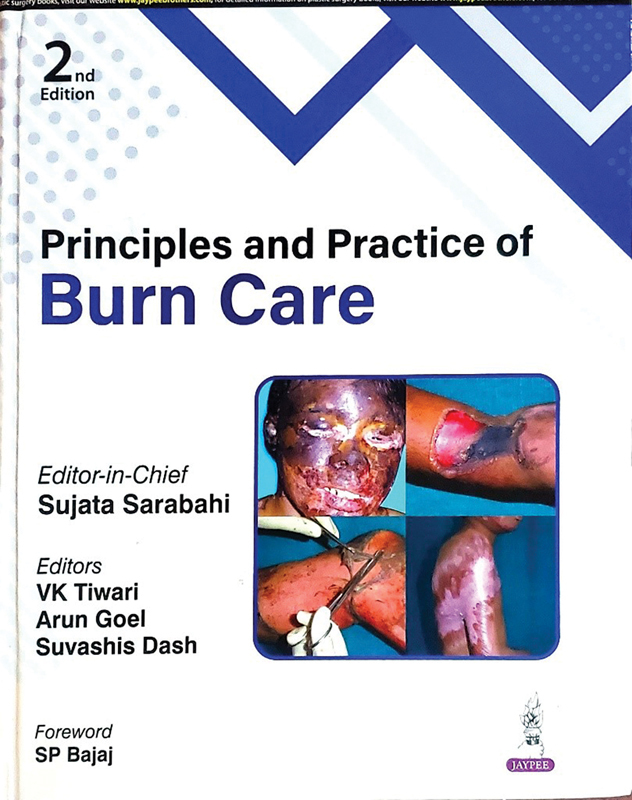
Principles and Practice of Burn Care Cover Page.

The book was officially released during APSICON 2024 in Coimbatore, where I received the hard copy from Dr. Sujata ( Fig. 1 ). At first glance, the book's high-quality production by the renowned Jaypee Brothers is immediately striking, and as you flip through its pages, you realize it is an endearing book. The superior paper quality, sharp and clear photographs, and effective use of color in text and tables enhance its readability and appeal. These features make it not just a textbook but also an excellent atlas of burn care.

I had the opportunity to refer to the earlier edition of the textbook. The new book is organized into seven sections, mirroring the structure of the first edition but enriched with updated content and expanded chapters. The number of contributors has increased significantly from 34 to 58, and the material has grown by 108 pages, reflecting the inclusion of new advancements and perspectives.

Retaining the basic structure of the first edition, the comprehensive seven sections include general considerations and fundamentals of early care in burns, wound management, burns of special types and sites, later care, rehabilitation and reconstruction, and finally, organization of the burn unit and planning for burn disasters.

## Section Highlights

### General Considerations and Fundamentals

The treatment of burns has evolved greatly over the last several decades with greater understanding of fundamentals of pathophysiology. Accordingly, the foundational chapters have been updated to include the current evidence in burn shock pathophysiology, wound healing, microbiology, and immunology. Flowcharts and color figures are thoughtfully incorporated to aid the easy comprehension of readers.

### Early Care in Burns

This section essentially covers the initial evaluation, resuscitation, and monitoring. It also provides noteworthy updates in respiratory and renal system management with up-to-date definitions, technological advances, and therapeutic measures, with contributions from expertise from other medical specialties.

### Burn Wound Management

Section 3 has six chapters devoted to burn wound management and one on anesthesia. Several newer materials for burn wound coverage have become available over the past 15 years, and they have been incorporated in appropriate chapters. The use of cadaveric skin allograft in India has increased significantly since the first edition, and the skin banking chapter has comprehensively covered all the necessary details.

### Burns of Special Types and Sites

The burns have traditionally been categorized into “special types” and “special sites” and are covered in detail in Sections 4 and 5, respectively. All chapters on the special types, including electrical, chemical, radiation, frictional, and firecracker burns, have been updated with newer advances. The chapter on electrical burns has included several newer details and clarified controversies. The chapter on firecracker injuries covers all the aspects from ancient history to “green-crackers,” an important topic that is missing in all the burn books even today. Chapters that focus on specific hands, faces, and external genitalia have been thoroughly updated and revised, as have chapters on pediatric burns and burns during pregnancy.

### General Management and Rehabilitation

Section 6 deals with important chapters on management and rehabilitation. The chapters on MODS in burns and physical therapy have been written by a completely different set of authors with current updates. The chapter on postburn sequelae and their management is the largest chapter in the book, and it is rightfully so, emphasizing rehabilitation. Unless the various disfigurements and deformities of a burn victim can be treated well, mere survival is of no consequence. This chapter has made an effort to answer all the questions asked by students on their final practical exam and by clinicians in planning and treating patients with multiple sequelae. The update of newer guidelines in a chapter on medicolegal aspects provides a comprehensive overview of medicolegal and compensation issues related to burn injuries.

### Organization of Burn Care

The last section on burn disasters and the organization of burn units has been written and updated with beautiful details. It is indeed timely information, as a large number of new units are coming up in the country, both in the government and the private sector, and the increasing number of disasters involving multiple burn victims.

Lastly, I am quite impressed with the wealth of information in the appendices, including many ready-to-reckons such as the composition of intravenous fluids, body fluids, normal biochemical values, nutritive values of Indian food, nutrition assessment charts, etc.

## Strengths and Recommendations

The editors and authors have succeeded in creating a textbook that is both authoritative and practical. It is a treasure trove of knowledge for trainees, practicing surgeons, and health care providers in burn care. The multidisciplinary approach, enriched with clinical insights, makes it indispensable. Complex concepts are simplified by flowcharts, updated guidelines, and vivid illustrations, which ensure that the content is accessible and actionable.

I strongly recommend Principles and Practice of Burn Care (second edition) to MCh/DNB trainees, plastic surgeons, and all professionals involved in burn management. It is a valuable addition to any medical library, not just in India but across the developing world. Congratulations to the editors and contributors for this outstanding contribution to the field.
